# Association of sleep disturbance with calcitonin, disease severity and health index among patients with ankylosing spondylitis

**DOI:** 10.1097/MD.0000000000026934

**Published:** 2021-08-13

**Authors:** Chun-Hsiung Chen, Hung-An Chen, Hsien-Tzung Liao, Chen-Hung Chen

**Affiliations:** a Division of Allergy, Immunology and Rheumatology, Taipei Tzu Chi Hospital, Buddhist Tzu Chi Medical Foundation, New Taipei City, Taiwan; b School of Medicine, Tzu Chi University, Hualien, Taiwan; c Chi Mei Medical Center, Tainan, Taiwan; d National Yang-Ming University, and Taipei Veterans General Hospital, Taipei, Taiwan.

**Keywords:** ankyloisng spoindylitis, calcitonin, disease activity, health index, sleep disturbance

## Abstract

To investigate the association of sleep disturbance with calcium regulatory hormones, disease severity and health index among the patients with ankylosing spondylitis (AS).

There were 104 AS patients enrolled in the cross-sectional study, and their sleep quality was recorded. Serum levels of calcium, parathyroid hormone, vitamin D3 and calcitonin were measured. We evaluated patient's disease activity, functional ability, patient's global assessment, physical mobility, radiographic damage and health index. Blood ESR and CRP levels were tested.

Sleep quality was positively correlated with serum calcitonin levels (*r* = 0.260, *P* = .008). Bad sleep and advanced radiographic damage were found among the AS patients with detectable serum calcitonin levels (*P* < .05). Sleep quality was significantly correlated with disease duration, CRP, BASDAI, ASDAS-ESR, ASDAS-CRP, BASFI, BAS-G, BASMI and ASAS-HI among the AS patients (all *P* < .05). Female gender, longer disease duration, higher ASDAS-CRP and serum calcitonin levels (OR [95% CI] = 3.210 [1.012–10.181], *P* = .048) were independent factors associated with bad sleep. Inflammation, disease activity, functional ability, patient's global assessment and cervical rotation were useful in predicting bad sleep among the AS patients, and ASDAS-CRP was the best predictor (AUC = 0.772, *P* < .001).

Serum calcitonin levels was elevated in the AS patients with bad sleep, and may participate in the pathophysiology of sleep disturbance. Bad sleep was associated with female gender, longer disease duration, higher inflammation, disease activity, functional impairment, mobility restriction, poor patient's global assessment and health index in AS. ASDAS-CRP was best in predicting bad sleep.

## 1 Introduction

Spondyloarthritis (SpA) includes axial and peripheral SpA, characterized by inflammatory back pain, sacroiliitis, peripheral arthritis, enthesitis and dactylitis. It contains ankylosing spondylitis (AS), reactive arthritis, psoriatic arthritis, enteropathic spondyloarthritis, Juvenile-onset spondyloarthritis and undifferentiated spondyloarthritis.^[1]^ The inflammatory process can also involve extra-articular organs, causing uveitis, psoriasis and Crohn's disease/ulcerative colitis. AS is the most common disease among the SpA family. AS occurs predominantly in young males and the major clinical symptom is the chronic low back with morning stiffness. Long-term inflammation of the spines may result in the formation of syndesmophyte and the subsequent ankylosis of adjacent vertebral bodies, leading to progressive restriction of spinal mobility, impaired functional ability, risk of spinal fracture and poor health status.^[2]^

Sleep disturbance is a common health issue in the general population and also affects life quality in the AS patients. Presence of sleep disturbance was higher in AS patients than common people.^[3]^ Vitamin D3 enhances the intestinal calcium and phosphate absorption, and modulates osteoblasts and osteoclasts in the skeleton. Parathyroid hormone increases the concentration of calcium in the blood plasma. Calcitonin can inhibit osteoclasts function and decrease serum calcium level. However other physiology function of calcitonin still remains unclear. One animal research reported that calcitonin could affect the sleep cycle.^[4]^ To our knowledge, the association of sleep quality with serum calcitonin level has not yet been reported in humankind. Some studies reported that sleep quality was associated with disease severity in the AS patients.^[5–11]^ In this study, we aim to test serum calcium level and it's regulatory molecules, the parathyroid hormone, vitamin D3 and calcitonin in the AS patients, and investigate their association with sleep quality. We evaluated the sleep quality among the AS patients and assessed the association with systemic inflammation, disease activity, functional ability, patient's global assessment, physical mobility, radiographic damage and health index.

## 2 Methods

### 2.1 Patients

We collected 104 AS patients who fulfilled the 1984 modified New York criteria^[12]^ and visited the outpatient department of Taipei Tzu Chi Hospital in Taiwan from August 1, 2016 to June 30, 2017. Blood samples were obtained from the AS patients. This study was conducted in compliance with the Declaration of Helsinki and Good Clinical Practice Guidelines established by the International Conference Harmonisation. This research was approved by the Institutional Review Board of Taipei Tzu Chi Hospital, Taiwan (04-XD14-054). Written informed consent was obtained from all the participants before study.

### 2.2 Sleep quality

Patient's overall sleep quality was recorded and answered the following question. During the past month, how would you rate your sleep quality overall with 4 categories: very good, fairly good, fairly bad and very bad sleep, which were scored ranging from 0 to 3, respectively.^[13]^ The patients with very good and fairly good sleep score were grouped as having good sleep quality. The patients with very bad and fairly bad sleep scores were grouped as having bad sleep quality.

### 2.3 Immunoassay of serum parathyroid hormone, vitamin D3, calcitonin and calcium

Samples of peripheral blood were obtained by venipuncture, allowed to complete clot formation, and the serum was centrifuged at 1710×*g* (3000 rpm) for 10 minutes and separated from the clot as soon as possible. Measurement of serum concentrations of intact parathyroid hormone (iPTH) were performed with a commercial quantitative chemiluminescent immunoassay (CLIA) kits (Siemens” Reagents for Intact PTH Assay). The iPTH assay is a 2-site sandwich immunoassay using direct chemiluminometric technology to measure iPTH in human serum and EDTA-plasma. Measurement of serum concentrations of 25-hydroxyvitamin D [25(OH)D3] were performed with a commercial quantitative CLIA kits (Diasorin, LIAISON 25OH Vitamin D TOTAL Assay [310600]). The 25-hydroxyvitamin assay is a direct competitive CLIA for quantitative determination of total 25OHD3 in serum and EDTA-plasma. Measurement of serum concentrations of calcitonin were performed with a commercial quantitative CLIA kits (IMMULITE 2000, Calcitonin). The calcitonin assay is a solid-phase, enzyme-labeled, 2-site chemiluminescent immunometric assay to measure calcitonin in human serum and EDTA-plasma. Measurement of serum levels of iPTH, 25-hydroxyvitamin D and calcitonin was done according to the manufacturer's instructions. The iPTH assay limit of ranged from 2.5 to 1900 pg/ml. The 25-hydroxyvitamin D assay limit of ranged from 4.0 to 150 ng/ml. The calcitonin assay limit of range: Males: Absolute range: ND–18.2 pg/ml, Females: Absolute range: ND–11.5 pg/mL. The serum calcitonin level **<**2 pg/ml was non-detectable. Measurement of serum calcium level was performed in the central laboratory in Taipei Tzu Chi Hospital, Taiwan (SIEMENS Dimension clinical chemistry system, calcium). The expected serum calcium level ranged from 2.12 to 2.52 mmol/l.

### 2.4 Disease activity, functional ability, patient's global assessments, physical mobility and systemic inflammation

We evaluated patient's disease severity, included disease activity, functional ability and patient's global assessments by using the Bath Ankylosing Spondylitis Disease Activity Index (BASDAI),^[14]^ Bath Ankylosing Spondylitis Functional Index (BASFI)^[15]^ and Bath Ankylosing Spondylitis Patient Global Score (BAS-G).^[16]^ Physical examinations were performed to determine the patient's physical mobility, including tragus-to-wall distance, lumbar flexion (Modified Schober test), intermalleolar distance, cervical rotation, lateral lumbar flexion, fingertip-to-floor distance, chest expansion and occiput-to-wall distance. The first 5 physical parameters constitute the Bath Anklyosing Spondylitis Metrology Index (BASMI).^[17]^ The BASDAI, BASFI, BAS-G and BASMI scores had a range from 0 to 10, and the higher scores of each parameters indicated more severe disease. Systemic inflammation markers, ESR and CRP levels, were tested and calculated the disease activity, including Ankylosing Spondylitis Disease Activity Index with ESR (ASDAS-ESR) and Ankylosing Spondylitis Disease Activity Index with CRP (ASDAS-CRP).^[18,19]^

### 2.5 Radiographic damage

Radiographs were taken in these AS patients including anteroposterior and lateral cervical/lumbar spine, pelvis and hip. The sacroiliac joints were scored according to the modified New York criteria.^[20]^ The severity of radiological damage in the cervical spines, lumbar spine and hip joint were assessed by the Bath Ankylosing Spondylitis Radiology Index (BASRI), and modified Stoke Ankylosing Spondylitis Spinal Score (m-SASSS).^[21,22]^ The total BASRI score ranged from 2 to 16, and the m-SASSS score ranged from 0 to 72.

### 2.6 Health index and depression

We assessed these AS patient's health status by using Ankylosing Spondylitis Disease Activity Index Health Index (ASAS-HI). The ASAS-HI includes 17 items which cover most of the International classification of functioning, disability and health (ICF) core set, score range from 0 to 17.^[23]^ The ASAS-HI covers these categories in AS patients: pain, maintaining a body position, moving around running, toileting, energy and drive, motivation, sexual functions, driving, community life, moving around walking, handing stress, recreation and leisure, emotional functions, washing oneself, economic self-sufficiency, sleep and handling stress. We use the ASAS-HI Taiwanese version to assess these AS patients.^[24]^

We also evaluated patient's mood of depression with a numerical rating scale, ranging from 0 to 10, and the higher score indicated more depressive condition.

### 2.7 Statistical analysis

Statistical analyses were carried out using the SPSS statistical package (SPSS for Windows, Chinese Version 10.0.7C, SPSS Inc., 2000). Clinical and laboratory variables were not in normal distribution. Correlations between sleep quality and variables were determined by using the Spearman's Rank Correlation test. To compare variables between the patients with non-detectable and detectable serum calcitonin level, Mann–Whitney *U* test was used. We then used Multivariate logistic regression analysis to calculate the odds ratios (OR) and 95% confidence intervals of variables for prediction of bad sleep quality (fairly bad and vary bad sleep). Receiver operating characteristic (ROC) curve analysis was used to evaluate and compare the performance of each clinical index in predicting the patients with bad sleep quality (fairly bad and very bad sleep). *P* values were regarded as being significant if they were less than .05.

## 3 Results

### 3.1 Demographics and clinical features

The demographics and clinical characteristics of the 104 AS patients are shown in Table 1. Among the 104 AS patients, 7 had very good sleep, 59 had fairly good sleep, 36 had fairly bad sleep and 2 had very bad sleep. 63.5% (66/104) patients had good sleep quality and 36.5% (38/104) patients had bad sleep quality.

**Table 1:** Demographic and clinical features of the 104 AS patients.

Characteristic	Total AS patients (n = 104)
Male/female	90/14
Age (yr)	45.981 (12.157)
Onset age (y/o)	26.980 (10.421)
Disease duration (yr)	19.157 (11.177)
HLA-B27 (+)	98/104
Sleep score (range: 0–3)	1.317 (0.627)
ESR (mm/h)	13.267 (12.764)
CRP (mg/dl)	0.709 (1.031)
BASDAI	2.685 (1.827)
ASDAS-ESR	1.941 (0.885)
ASDAS-CRP	2.057 (0.983)
BASFI	1.099 (1.415)
BAS-G	3.382 (2.801)
BASMI	3.257 (1.930)
Tragus-to-wall distance (cm)	15.420 (5.397)
Modified Schober index (cm)	4.307 (1.798)
Intermalleolar distance (cm)	114.620 (23.107)
Cervical rotation (°)	63.750 (29.262)
Lateral lumbar flexion (cm)	11.048 (6.918)
Fingertip-to-floor distance (cm)	19.168 (13.469)
Chest expansion (cm)	2.471 (1.473)
Occiput-to-wall distance (cm)	6.620 (6.136)
BASRI-Total	9.849 (2.590)
m-SASSS	31.051 (18.135)
ASAS-Health index (HI)	4.917 (3.337)
Depression (range: 0–10)	1.513 (1.964)

### 3.2 Correlation between sleep quality and serum parameters among the 104 AS patients

Correlation between sleep quality and serum parameters among the AS patients were determined by the Spearman's Rank Correlation test. Interestingly, sleep score was correlated with the serum calcitonin level (*r* = 0.260, *P* = .008) in our study (Table 2). But sleep score was not correlated with serum levels of calcium, iPTH and 25-hydroxyvitamin D. This result suggested that sleep disturbance was positively associated with serum calcitonin level in AS. Sleep disturbance was not associated with serum parathyroid hormone, vitamin D3 and calcium level.

**Table 2:** Correlations between sleep quality and various parameters among the 104 AS patients.

Clinical parameters	Sleep score
Age (yr)	0.119 (.231)
Onset age (y/o)	0.002 (.987)
Disease duration (yr)	0.196 (.048^∗^)
Calcium	0.127 (.200)
Intact parathyroid hormone (iPTH)	0.004 (.966)
Vitamin D3 (25-hydroxyvitamin D)	0.005 (.963)
Calcitonin	0.260 (.008^∗^)
ESR (mm/h)	0.100 (.322)
CRP (mg/dl)	0.204 (.040^∗^)
BASDAI	0.390 (<.001^∗^)
ASDAS-ESR	0.378 (<.001^∗^)
ASDAS-CRP	0.394 (<.001^∗^)
BASFI	0.325 (.001^∗^)
BAS-G	0.306 (.002^∗^)
BASMI	0.210 (.037^∗^)
Fingertip-to-floor distance	0.124 (.208)
Chest expansion	−0.104 (.293)
Occiput-to-wall distance	0.131 (.184)
BASRI-Total	0.081 (.426)
m-SASSS	0.146 (.150)
ASAS-Health index (HI)	0.311 (.001^∗^)
Depression	0.192 (.052)

### 3.3 Correlation between sleep quality and clinical parameters among the 104 AS patients

Correlation between sleep quality and clinical parameters were determined by the Spearman's Rank Correlation test, and are shown in Table 2. Sleep score was correlated with disease duration (*r* = 0.196, *P* = .048) and CRP level (*r* = 0.204, *P* = .040), suggesting that sleep disturbance was positively associated with longer disease duration and systemic inflammation in AS. Sleep score was correlated with BASDAI (*r* = 0.390, *P* < .001), ASDAS-ESR (*r* = 0.378, *P* < .001) and ASDAS-CRP (*r* = 0.394, *P* < .001). Among the BASDAI individual parameters, sleep score was correlated with BASDAI-1 (*r* = 0.282, *P* = .004), BASDAI-2 (*r* = 0.392, *P* < .001), BASDAI-5 (*r* = 0.297, *P* = .002) and BASDAI-6 (*r* = 0.272, *P* = .005) (not shown in Table 2). These results suggested that sleep disturbance was positively associated with patient's disease activity, particularly the degree of fatigue, back pain and morning stiffness.

Sleep score was correlated with the BASFI (*r* = 0.325, *P* = .001). Among the individual BASFI parameters, sleep score was correlated with BASFI-8 (*r* = 0.254, *P* = .010) and BASFI-10 (*r* = 0.210, *P* = .033) (not shown in Table 2). Sleep disturbance was positively associated with functional ability, particularly the function of looking over your shoulder without turning your body and doing a full day activities whether it be at home or work. Sleep score was correlated with BAS-G (*r* = 0.306, *P* = .002). Among the individual BAS-G parameters, sleep score was correlated BAS-G-1 (*r* = 0.327, *P* = .001) (not shown in Table 2). Sleep disturbance was positively associated with patient's global assessment, particularly how have you been over the last week.

Sleep score was correlated with BASMI (*r* = 0.210, *P* = .037). Among the individual BASMI parameters, sleep score was correlated with cervical rotation (*r* = −0.227, *P* = .023) and lateral lumbar flexion (*r* = −0.213, *P* = .030) (not shown in Table 2). These results suggested that sleep disturbance was positively associated with patient's physical mobility, particularly the degree of cervical rotation and lateral lumbar flexion. Sleep score was correlated with ASAS-HI (*r* = 0.311, *P* = .001), suggesting that sleep disturbance was positively associated with patient's poor health status.

Sleep score did not show significant correlation with age, onset age, ESR, depression score, BASRI-Total and m-SASSS in our study.

### 3.4 Comparison of clinical parameters between the AS patients with non-detectable and detectable serum calcitonin level

We compared the clinical parameters between the AS patients with non-detectable (<2 pg/ml) and detectable (≧2 pg/ml) serum calcitonin level by using Mann–Whitney *U* test (Table 3). BASRI-Total (10.356 [2.579] vs 9.287 [2.510], *P* = .040) and m-SASSS (35.250 [18.534] vs 26.404 [16.666], *P* = .014) were significantly higher in the detectable than non-detectable group. Sleep score (1.455 [0.603] vs 1.163 [0.624], *P* = .031) was significantly higher in the detectable than non-detectable group. The patients with detectable serum calcitonin level had advanced radiographic damage and bad sleep. Age, onset age, disease duration, ESR, CRP, BASDAI, ASDAS-ESR, ASDAS-CRP, BASFI, BAS-G, BASMI, ASAS-HI and depression did not show difference between the detectable and non-detectable group.

**Table 3:** Comparison of clinical parameters between the AS patients with non-detectable and detectable serum calcitonin level.

	Serum calcitonin level		
Parameters	Non-detectable (<2 pg/ml)	Detectable (≧2 pg/ml)	*P* values
Age (yr)	43.959 (12.393)	47.782 (11.763)	.125
Onset age (y/o)	27.479 (11.357)	26.537 (9.599)	.989
Disease duration (yr)	16.958 (10.433)	21.111 (11.544)	.058
Sleep score	1.163 (0.624)	1.455 (0.603)	.031^∗^
ESR (mm/h)	14.104 (13.244)	12.509 (12.391)	.690
CRP (mg/dl)	0.656 (0.850)	0.759 (1.179)	.524
BASDAI	2.852 (1.942)	2.534 (1.725)	.504
ASDAS-ESR	1.972 (0.848)	1.914 (0.923)	.785
ASDAS-CRP	2.021 (0.862)	2.090 (1.088)	.986
BASFI	0.920 (1.328)	1.257 (1.483)	.135
BAS-G	3.117 (2.379)	3.618 (3.133)	.686
BASMI	2.887 (1.625)	3.577 (2.124)	.169
BASRI-Total	9.287 (2.510)	10.356 (2.579)	.040^∗^
m-SASSS	26.404 (16.666)	35.250 (18.534)	.014^∗^
ASAS-HI	4.948 (3.072)	4.889 (3.584)	.609
Depression	1.438 (2.133)	1.578 (1.822)	.376

### 3.5 Multivariate logistic regression analysis of sleep disturbance associated with clinical and serum parameters among the 104 AS patients

In multivariate logistic regression analysis (Table 4), bad sleep showed significant association with gender (OR [95% CI] = 0.134 [0.027–0.654], *P* = .013), disease duration (OR [95% CI] = 1.071 [1.017–1.128], *P* = .009), ASDAS-CRP (OR [95% CI] = 4.501 [2.237–9.055], *P* < .001) and serum calcitonin level (OR [95% CI] = 3.210 [1.012–10.181], *P* = .048). These results suggested that female gender, longer disease duration, higher disease activity and serum calcitonin level were independent factors associated with bad sleep among the AS patients.

**Table 4:** Multivariate logistic regression analysis of bad sleep associated with gender, disease duration, disease activity and serum calcitonin level among the 104 AS patients.

	OR (95% CI)	*P* value
Bad sleep (fairly bad + very bad)		
Gender	0.134 (0.027–0.654)	.013^∗^
Disease duration	1.071 (1.017–1.128)	.009^∗^
ASDAS-CRP	4.501 (2.237–9.055)	<.001^∗^
Calcitonin^†^	3.210 (1.012–10.181)	.048^∗^

### 3.6 ROC curve analysis to assess the usefulness of clinical parameters in predicting the AS patients with bad sleep quality

**Figure 1:**
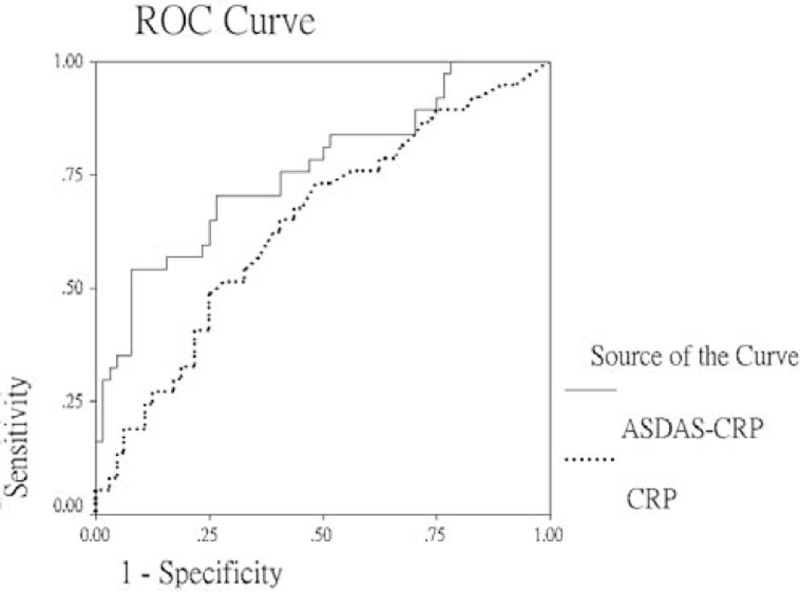
Receiver operating characteristic (ROC) plot analysis: Using ASDAS-CRP and CRP to predict the AS patients with bad sleep (fairly bad + very bad). The AUC (*P* value) are .772 (<.001) and .675 (.006) for ASDAS-CRP and CRP, respectively. AUC = area under the curve.

We used ROC curve analysis to evaluate the degree of usefulness of clinical parameters to predict the AS patients with bad sleep quality (fairly bad and very bad sleep). The parameters which showed significant in predicting bad sleep quality among the AS patients were CRP level (Area under the curve [AUC] = 0.675, *P* = .006), BASDAI [AUC = 0.753, *P* < .001], ASDAS-ESR [AUC = 0.759, *P* < .001], ASDAS-CRP [AUC = 0.772, *P* < .001], BASFI [AUC = 0.694, *P* = .002], BAS-G [AUC = 0.711, *P* = .001] and cervical rotation [AUC = 0.364, *P* = .033]. Disease duration, ESR, BASRI-Total and m-SASSS did not show significance in predicting bad sleep among the AS patients. These results suggested that systemic inflammation, disease activity, functional ability, global assessment, cervical rotation, and serum calcitonin level could predict patient's sleep disturbance in AS. The disease activity score, particular ASDAS-CRP, showed the highest AUC values (0.772) in predicting bad sleep quality among the AS patients. Figure 1 shows using ASDAS-CRP and CRP to predict the AS patients with bad sleep (fairly bad + very bad).

## 4 Discussion

### 4.1 Sleep associated with calcitonin

In our study, 63.5% (66/104) patients had good sleep quality and 36.5% (38/104) patients had bad sleep quality. Previous studies showed that the prevalence of sleep disturbance was higher in the AS patients than healthy control.^[2,6,8,24–26]^ Interestingly, sleep score was significantly correlated with the serum calcitonin level, but not with calcium, iPTH and 25-hydroxyvitamin D level. Serum calcitonin level was an independent factor associated with bad sleep in AS. Sleep score was significantly higher in the patient with detectable than non-detectable serum calcitonin level. Radiographic damage, the BASRI-Total and m-SASSS were significantly higher in the patients with detectable than non-detectable serum calcitonin level. Higher serum calcitonin level was associated with bad sleep and advanced radiographic change in the AS patients. Multiple testing Bonferroni corrections were not performed in the study, because it may interpret the results too conservatively to miss some new valuable findings.

Our study suggested that serum calcitonin may be a potential therapeutic target for sleep disturbance. Slisli Y et al showed that salmon calcitonin is an extremely potent suppressor of slow wave sleep, producing an almost 30-h long insomnia.^[4]^ Carman JS et al showed that when synthetic salmon calcitonin given in the evening, it appeared to delay sleep onset.^[27]^ On the contrary, Nakajima Te al showed that intramuscular calcitonin improved depth and maintenance of sleep in a case report.^[28]^ Serum calcitonin level correlated with bad sleep, and may have influence on sleep quality. But the causative relationship between calcitonin and bad sleep needs further investigation.

### 4.2 Sleep associated with female gender, disease duration, and disease activity

Our study showed that sleep score was correlated with disease duration, but not with age in the AS patients. Disease duration was an independent factor associated with bad sleep quality in the AS patients. In contrast, Li Y et al showed that the PSQI score was associated with age, but was not associated with disease duration in AS.^[26]^ Our study suggested that female gender was an independent factor associated with bad sleep quality in the AS patients, compatible with previous studies female patient had more sleep disturbance.^[5,7]^ Female gender and longer disease duration may be associated with more sleep disturbance in the AS patients.

Our study showed that CRP level was correlated with sleep score, and was a predictor for bad sleep quality among the AS patients. Disease activity including BASDAI, ASDAS-ESR and ASDAS-CRP were correlated with sleep score. All the 3 disease activity parameters were predictors for bad sleep quality among the AS patients. Disease activity was an independent factor associated with bad sleep. ASDAS-CRP showed the highest AUC value (0.772) in predicting bad sleep among the AS patients. Fatigue (BASDAI-1), back pain (BASDAI-2) and morning stiffness (BASDAI-5 and -6) were particularly associated with bad sleep. Aydin et al showed that there was a positive correlation between the BASDAI and PSQI scores in AS patients.^[8]^ Li et al showed that PSQI score was associated with ESR, CRP, BASDAI, pain and morning stiffness in AS.^[26]^ Wadeley et al showed that poor sleep was associated with greater disease activity, specifically, spinal pain, stiffness and fatigue.^[5]^ Zhang et al showed that sleep quality was positively correlated with nocturnal pain.^[9]^ Hakkou et al showed that higher disease activity and pain were present in patients with sleep disturbance.^[11]^ Anti-TNF agents could improve sleep quality in active AS patients.^[29–31]^ Systemic inflammation and patient's disease activity, especially fatigue, back pain and morning stiffness, were associated with bad sleep quality in the AS patients. Greater disease activity, particularly the ASDAS-CRP, was the most important predictor for sleep disturbance among the AS patients. Aggressive treatment of disease activity to target, may be helpful to improve sleep quality in the AS patients. Evidence suggests that sleep and pain are reciprocally related.^[32,33]^ Chronic pain may impair a patient's life quality including sleep disturbance. Sleep disturbance may contribute to the development and maintenance of chronic pain.

### 4.3 Sleep associated with functional ability, physical mobility and global assessment

The BASFI, particularly the BASFI-8 (Looking over your shoulder without turning body) and BASFI-10 (Doing a full day activities whether it be at home or at work), were correlated with sleep score in our study. The functional ability was a predictor for bad sleep among the AS patients. Higher BASFI was presented in the patients with poorer sleep quality in AS.^[6,7,11]^ Da Costa D et al showed that worse functional status was associated with poorer sleep quality, longer sleep latency, shorter sleep duration and poorer sleep efficiency.^[34]^ Poor functional ability was associated with bad sleep quality in the AS patients. Maintaining good functional ability may be helpful for sleep quality in the AS patients.

The BASMI, particularly the cervical rotation and lateral lumbar flexion were correlated with sleep score in our study. Cervical rotation was a predictor for bad sleep quality among the AS patients. Sariyildiz MA et al showed that poor sleep quality was positively correlated with mobility restrictions. The lower quality of sleep is greatly associated with increased limitation of mobility.^[10]^ However, Li Y et al showed that the PSQI score were not associated with BASMI.^[26]^ Poor physical mobility, particularly cervical rotation and lateral flexion were associated with bad sleep quality in the AS patients. Maintaining good physical mobility may be helpful for sleep quality in the AS patients.

The BAS-G and BAS-G1 were correlated with sleep score in our study, and BAS-G was a predictor for bad sleep quality in the AS patients. Patient's health index was correlated with sleep score in our study. Li et al showed that the PSQI score was associated with overall assessment of health in AS.^[26]^ Sariyildiz et al showed that poor sleep quality positively correlated with poor quality of life. The lower quality of sleep is greatly associated with quality of life.^[10]^ Leverment et al showed that disturbed sleep associated with quality of life in AS.^[6]^ Poor patient's global assessment and health index were related to bad sleep in the AS patients.

This study has some limitations. More clinical data that might be the confounding factor need to be collected, including patient's comorbidity and medication. Our study supports the association of serum calcitonin level with sleep quality, but it cannot be established on such a small cohort. A larger-scale study using the Pittsburgh Sleep Quality Index (PSQI) score to assess the association of sleep quality with serum calcitonin level and disease severity is needed. The serum calcitonin level in normal population and it's relation with sleep quality requires further investigation.

## 5 Conclusions

Elevated serum calcitonin level was found in the AS patients with bad sleep, and may participate in the pathophysiology of sleep disturbance. Sleep disturbance was associated with female gender, longer disease duration, higher inflammation, disease activity, functional impairment, mobility restriction, poor patient's global assessment and health index in the AS patients. Fatigue, back pain, morning stiffness and cervical rotation were particularly associated with bad sleep. ASDAS-CRP is the most useful predictor for bad sleep among the AS patients.

## Acknowledgments

We thank Lin-Lan Lin and Chun-Wei Chen for obtaining questionnaires and performing physical examination.

## Author contributions

**Conceptualization:** Chun-Hsiung Chen, Hung-An Chen.

**Data curation:** Chun-Hsiung Chen, Chen-Hung Chen.

**Formal analysis:** Chun-Hsiung Chen.

**Funding acquisition:** Chun-Hsiung Chen.

**Investigation:** Chun-Hsiung Chen, Chen-Hung Chen.

**Methodology:** Chun-Hsiung Chen, Hsien-Tzung Liao.

**Writing – original draft:** Chun-Hsiung Chen.

**Writing – review & editing:** Chen-Hung Chen, Hung-An Chen, Hsien-Tzung Liao.
